# Fecal levels of SCFA and BCFA during capecitabine in patients with metastatic or unresectable colorectal cancer

**DOI:** 10.1007/s10238-023-01048-7

**Published:** 2023-04-07

**Authors:** Janine Ziemons, Romy Aarnoutse, Anne Heuft, Lars Hillege, Janneke Waelen, Judith de Vos-Geelen, Liselot Valkenburg-van Iersel, Irene E. G. van Hellemond, Geert-Jan M. Creemers, Arnold Baars, Johanna H. M. J. Vestjens, John Penders, Koen Venema, Marjolein L. Smidt

**Affiliations:** 1 GROW - School for Oncology and Reproduction, Maastricht University, Maastricht, The Netherlands; 2 Department of Surgery, Maastricht University Medical Centre, Maastricht, The Netherlands; 3 Department of Internal Medicine, Division of Medical Oncology, Maastricht University Medical Centre, Maastricht, The Netherlands; 4 Department of Medical Oncology, Catharina Hospital, Eindhoven, The Netherlands; 5 Department of Medical Oncology, Hospital Gelderse Vallei, Ede, The Netherlands; 6 Department of Internal Medicine, VieCuri Medical Centre, Venlo, The Netherlands; 7 NUTRIM - School of Nutrition and Translational Research in Metabolism, Maastricht University, Maastricht, The Netherlands; 8 Department of Medical Microbiology, Infectious Diseases and Infection Prevention, Maastricht University Medical Centre, Maastricht, The Netherlands; 9 Euregional Microbiome Center, Maastricht, The Netherlands; 10 Centre for Healthy Eating and Food Innovation, Maastricht University – Campus Venlo, Venlo, The Netherlands

**Keywords:** Gut microbiota, Palliative chemotherapy, Tumor response, Chemotherapy toxicity, Inflammation

## Abstract

**Background:**

Gut bacteria-derived short-chain fatty acids (SCFA) and branched-chain fatty acids (BCFA) are considered to have beneficial metabolic, anti-inflammatory as well as anti-carcinogenic effects. Previous preclinical studies indicated bidirectional interactions between gut bacteria and the chemotherapeutic capecitabine or its metabolite 5-FU. This study investigated the effect of three cycles of capecitabine on fecal SCFA and BCFA levels and their associations with tumor response, nutritional status, physical performance, chemotherapy-induced toxicity, systemic inflammation and bacterial abundances in patients with colorectal cancer (CRC).

**Methods:**

Forty-four patients with metastatic or unresectable CRC, scheduled for treatment with capecitabine (± bevacizumab), were prospectively enrolled. Patients collected a fecal sample and completed a questionnaire before (T1), during (T2) and after (T3) three cycles of capecitabine. Tumor response (CT/MRI scans), nutritional status (MUST score), physical performance (Karnofsky Performance Score) and chemotherapy-induced toxicity (CTCAE) were recorded. Additional data on clinical characteristics, treatment regimen, medical history and blood inflammatory parameters were collected. Fecal SCFA and BCFA concentrations were determined by gas chromatography–mass spectrometry (GC–MS). Gut microbiota composition was assessed using 16S rRNA amplicon sequencing.

**Results:**

Fecal levels of the SCFA valerate and caproate decreased significantly during three cycles of capecitabine. Furthermore, baseline levels of the BCFA iso-butyrate were associated with tumor response. Nutritional status, physical performance and chemotherapy-induced toxicity were not significantly associated with SCFA or BCFA. Baseline SCFA correlated positively with blood neutrophil counts. At all time points, we identified associations between SCFA and BCFA and the relative abundance of bacterial taxa on family level.

**Conclusions:**

The present study provided first indications for a potential role of SCFA and BCFA during capecitabine treatment as well as implications for further research.

**Trial registration:**

The current study was registered in the Dutch Trial Register (NTR6957) on 17/01/2018 and can be consulted via the International Clinical Trial Registry Platform (ICTRP).

**Supplementary Information:**

The online version contains supplementary material available at 10.1007/s10238-023-01048-7.

## Background

In recent years, it has become increasingly evident that the gut microbiota plays a crucial role in the development, manifestation and treatment of different types of cancer. For instance, there is accumulating evidence that the gut microbiota interacts with chemotherapeutic drugs via various mechanisms [1–3]. In this context, the role of gut microbiota-derived metabolites such as short-chain fatty acids (SCFA) and branched-chain fatty acids (BCFA) is of particular interest. A proportion of these metabolites is absorbed into the bloodstream, where they can exert not only local but also systemic effects, in that way functioning as a linking factor between the gut microbiota and human metabolism as well as carcinogenesis [4, 5].

The SCFA acetate, propionate, butyrate, valerate and caproate are produced by gut bacteria through different metabolic pathways. In particular, dietary non-digestible carbohydrates are an important substrate for microbial fermentation and subsequent SCFA production [6]. SCFA have been shown to have pivotal effects on human metabolism and the immune system. For instance, several studies showed that SCFA have potent anti-inflammatory effects by among others inhibition of histone deacetylases (HDACs) and NF-κB as well as by interaction with several G-protein coupled receptors and modulation of cytokine production [4, 7, 8]. Particularly butyrate is essential for gut barrier function by serving as primary energy source for colonocytes and by modulating the expression of tight junction proteins and mucins [4, 8]. In addition, SCFA have been shown to have various effects on human macronutrient metabolism and metabolic health [9].

Preclinical studies also indicated direct anti-carcinogenic effects of SCFA, as well as the potential that SCFA could sensitize cancer cells to chemotherapeutic agents. For instance, Encarnação et al. observed anti-proliferative effects of butyrate and a synergistic effect of irinotecan and butyrate in different colon cancer cell lines [10]. Very recently, Kim et al. have described anti-carcinogenic effects of butyrate in colon cancer cell lines, which could be potentiated by the addition of a growth medium from *Lactiplantibacillus plantarum* in butyrate-resistant cells [11]. Furthermore, SCFA might reduce (gastrointestinal) side effects of the chemotherapy, which are commonly caused by intestinal barrier disruption and inflammation [12]. In support of this, previous research indicated that the administration of prebiotics, alone or in combination with probiotics, might reduce the occurrence of serious side effects of chemotherapy [13, 14]. Prebiotics are substrates that can be metabolized by several gut bacteria to produce SCFA, thereby stimulating the growth of SCFA-producing bacteria [15]. However, it should be noted that the literature concerning the physiological roles of SCFA is divergent since some studies also described pro-inflammatory or oncogenic properties under certain circumstances [3, 7].

In contrast to SCFA, there is currently only limited knowledge concerning the exact physiological roles of BCFA. The BCFA iso-butyrate and iso-valerate are produced by the gut microbiota from branched-chain amino acids or are directly ingested via the diet (e.g., through beef and milk products) [16, 17]. While BCFA are mostly studied in the context of the neonatal gut, evidence concerning physiological effects in the adult gut is scarce, but it has been suggested that BCFA also play a role in human energy metabolism and might have anti-inflammatory as well as anti-carcinogenic properties [16, 18]. On the other hand, branched-chain amino acids, the precursors of BCFA, are considered to play a role in insulin resistance, which could indicate a potentially negative effect on metabolic health [19].

In view of these physiological effects of SCFA and BCFA that are also relevant in the setting of cancer treatment, it might be expected that these microbial metabolites also play a role during treatment with chemotherapy, for instance capecitabine. Capecitabine is an orally administered prodrug that is converted intratumorally to the cytotoxic compound 5-fluorouracil (5-FU) and which is commonly administered in patients with metastatic colorectal cancer (mCRC) [20]. In the last years, there is increasing evidence for bidirectional interactions between 5-FU-based therapies and the gut microbiota. For instance, large-scale in vitro screening studies showed that capecitabine/5-FU did not only impact the growth of several bacterial species [21], but could also be metabolized by specific gut bacteria [22]. In addition, 5-FU induced shifts in gut microbiota composition in mice [23]. Furthermore, it has been indicated that *Fusobacterium nucleatum* might be able to induce chemoresistance to 5-FU in CRC cells, while *Lactiplantibacillus* (previously *Lactobacillus*) *plantarum*-derived supernatant seemed to sensitize CRC cells to the anticancer effects of 5-FU [24–26]. In contrast to this, our research group did not detect consistent capecitabine-induced changes in gut microbiota composition and diversity in a relatively small and heterogeneous group of CRC patients [27]. Other clinical studies, using different chemotherapeutics, described that chemotherapy treatment affected gut microbiota composition and the abundance of prominent SCFA-producing bacteria such as *Veillonella* and *Prevotella* [14, 28, 29]. While most of the previous research focused on the abundance of gut bacteria, more activity-based analyses, such as the measurement of microbial metabolites, would be of special interest in a clinical setting. Even if there was no major effect of capecitabine on taxa abundance in the previous study [27], metabolic activity and the production of relevant metabolites might have been changed in these patients, with possible clinical implications.

In mice, Ferreira et al. showed that oral administration of SCFA and particularly butyrate could counteract 5-FU-induced intestinal mucositis [30]. However, there is currently no knowledge of the role of gut microbiota-derived SCFA and BCFA during 5-FU-based chemotherapy in a clinical setting. The present research aims to fill this gap of knowledge and investigates the effect of three cycles of capecitabine on fecal SCFA and BCFA levels and their associations with tumor response, nutritional status, physical performance, chemotherapy-induced toxicity, as well as systemic inflammation in patients with metastatic or unresectable CRC. Based on previous studies and the described beneficial effects of SCFA, it might be expected that fecal SCFA levels would reduce during capecitabine. In addition, it is hypothesized that higher SCFA levels would be associated with better tumor response, a less fragile nutritional status, increased physical performance, less toxicity and reduced systemic inflammation. Furthermore, associations between fecal SCFA and BCFA levels and the abundance of microbial taxa are explored in this patient population.

## Methods

### Study design and patient inclusion

This prospective longitudinal multicenter cohort study was conducted in four hospitals in the Netherlands (Maastricht University Medical Center (MUMC +), Catharina Hospital Eindhoven, Hospital Gelderse Vallei, VieCuri Medical Center) between 2017 and 2020. Patients with metastatic and/or unresectable CRC who were planned for treatment with capecitabine (±intravenous VEGF inhibitor bevacizumab) were eligible for participation. Exclusion criteria were abdominal radiotherapy < 2 weeks before inclusion, other systemic therapy < 1 month before inclusion, antibiotic use < 3 months before inclusion, microsatellite instability (MSI-H) and impaired renal function (creatinine clearance of < 30 ml/min).

### Fecal sample collection

Fecal samples and questionnaires were collected before start of the first capecitabine cycle (T1), during the second week of the third cycle (T2) and after the third cycle (T3) (Figure S1). Each capecitabine cycle consisted of two weeks (days 1–14) oral capecitabine ingestion (2 × per day) and one week of rest (days 15–21). Patients were asked to collect the fecal samples at home in preservation-free tubes (*Sarstedt*) and to immediately store them in the freezer. The samples were transported to the hospital in a cooled container (*Sarstedt*) to prevent thawing and stored at − 20 °C for short-term and at − 80 °C for long-term storage.

### Clinical data collection

At the same time points, patients filled in a questionnaire concerning previous use of anti-, pre- or probiotics, medical history as well as nutritional status (Malnutrition Universal Screening Tool, MUST) and physical performance (Karnofsky Performance Score, KPS). The MUST scores the nutritional status on a scale between 0 (low risk) and 2 or more (high risk). The KPS is a scale between 0 and 100 (0: dead and 100: no physical complaints). The occurrence of chemotherapy-induced toxicity was scored based on the *Common Terminology Criteria for Adverse Events* (CTCAE, version 4.0) [31] and included scores on nausea, vomiting, diarrhea, constipation, peripheral sensory neuropathy, oral mucositis, hand–foot syndrome, fever, hair loss and fatigue (Table S1). Tumor response was evaluated based on CT or MRI scans which were performed before and at the end of three cycles of capecitabine. The tumor size change (%) was calculated as described in Table S2 and included as a continuous variable (with negative values indicating tumor decrease and positive values indicating tumor increase). In addition, RECIST (*Response Evaluation Criteria in Solid Tumours, version *1.1) was used to categorize tumor response as complete response, partial response, progressive disease or stable disease (Table S2) [32]. Additional data on clinical characteristics, treatment regimen, medical history as well as blood inflammatory parameters (leukocytes, neutrophils and thrombocytes) before the start of cycle 1 (around T1) and before the start of cycle 4 (around T3) were collected from medical records.

### Analysis of fecal levels of SCFA and BCFA

For SCFA/BCFA analysis, 500 mg of frozen fecal samples was mixed 1:1 (weight:weight) with PBS (5 min) and afterward centrifuged at 14.000 g for 10 min. Subsequently, 50 μl of supernatant was mixed with 650 μl internal standard solution, containing methanol, internal standard (2 mg/ml 2-ethyl butyric acid) and formic acid (20%). The SCFA/BCFA concentrations were determined through gas chromatography–mass spectrometry (GC–MS) *(8890 GC System, Agilent Technologies*) equipped with a PAL3 RSI 85 autosampler (*Agilent*). The temperature settings of the injector port, oven, flame ionization detector and mass spectrometer detector were 250 °C, 200 °C, 275 °C and 225 °C, respectively. In order to correct for sample consistency, measured SCFA and BCFA concentrations were divided by the sample dry weight (g). In order to assess sample dry weight, samples were weighed, freeze-dried until stable weight loss had occurred, and weighed again.

### Analysis of gut microbiota composition and bacterial abundances

Analysis of the gut microbiota was performed as previously described [27]. In short, metagenomic DNA from fecal samples was isolated using the Ambion MagMax™ Total Nucleic Acid Isolation Kit (*Thermo Fisher Scientific*). The manual preprocessing consisted of mechanical disruption with bead-beating, as well as chemical and thermal disruption. This was followed by automated nucleic acid purification with the KingFisher FLEX (*Thermo Fisher Scientific*). Upon PCR amplification of the 16S ribosomal RNA (rRNA) hypervariable V4 gene region, amplicons were sequenced on a MiSeq platform, as described by Galazzo et al. [33]. For preprocessing of the raw sequencing data, a standardized in-house pipeline using the software package DADA2 (R version 4.0.3) was applied [34].

### Statistical analysis of SCFA/BCFA levels and clinical variables

Baseline characteristics of the patient population were assessed using SPSS (version 27, IBM). All other statistical analyses were conducted using R in R Studio (R version 4.0.0) [35]. For all statistical tests, including procedures with correction for multiple testing, *p*-values < 0.05 were considered to be statistically significant. For continuous variables, the decision on normality was based on histograms, Q–Q plots and the Shapiro–Wilk test. For normally distributed data, the mean (± SD) is shown, while the median (± IQR) is shown if the assumption of normality was violated. For categorical variables, the number of patients (*n*) and percentages (%) are shown.

Longitudinal analysis of non-normal or ordinal variables was conducted using Friedman’s ANOVA and using complete cases (= patients who have measurements for all three time points) only. In case of significant results, post hoc analyses were performed by means of a paired Wilcoxon signed-rank test with Bonferroni correction using the *rstatix* package (version 0.7.0) [36]. For SCFA and BCFA, results were confirmed with linear mixed models by means of the *lmer* function from the *lme4* package (version 1.1-26), using log-transformed (log1p) data, sampling time point as fixed effect and patient ID as random effect [37].

For cross-sectional analyses comparing groups based on prior treatment, tumor response (RECIST) or capecitabine dose adjustments, Kruskal–Wallis or Mann–Whitney *U* test was used, depending on the number of groups to be compared. If the Kruskal–Wallis test showed significant results, post hoc analyses were performed using Dunn’s test with Bonferroni correction [38].

In order to analyze associations between SCFA/BCFA and clinical variables of interest, Spearman correlation was calculated using the *corr.test* function from the *psych* package (version 2.2.5) and a data frame with all variables of interest [39]. P-values were adjusted for multiple testing by means of false discovery rate (fdr) adjustment according to the Benjamini and Hochberg procedure [40]. Correlations with an adjusted *p*-value > 0.05 but < 0.07 are reported as a trend. Correlations between sample dry weight and diarrhea were calculated with *corr.test*, without fdr adjustment, since only those two variables were included. Visualization of correlations between SCFA/BCFA and clinical or blood inflammatory parameters was done by means of the *corrplot* package (version 0.92), using the correlation matrix from *corr.test* [41]. Scatterplots were made for all correlations between SCFA/BCFA and clinical or blood parameters that are reported in the article.

### Statistical analysis of gut microbiota data

Spearman correlations between fecal SCFA/BCFA and relative abundances of bacterial taxa on family level were calculated using the *corr.test* function from the *psych* package (version 2.2.5). Taxa present in less than 20% of the samples were filtered out for these analyses. P-values were adjusted for multiple testing by means of fdr adjustment. Correlations with an adjusted *p*-value > 0.05 but < 0.07 are reported as a trend. The correlation heatmaps were produced using the *cor_heatmap* function from the *microViz* package (version 0.9.2) and R version 4.1.3 [42]. For the correlation heatmaps, all taxa with *p*-value < 0.07 at one of the time points were included.

## Results

### Baseline clinical characteristics of the study population

In total, 44 patients with metastatic or unresectable CRC were included in the current study and completed the baseline sampling at T1. At T2, 38 fecal samples were collected, while 39 fecal samples were collected at sampling time point T3. Thirty-seven patients collected fecal samples at all three time points (Figure S1).

Of the total group, 45.5% received other systemic therapies before the start of capecitabine (> 1 month before inclusion) (Table 1). Prior systemic treatments included CAPOX (capecitabine + oxaliplatin) ± bevacizumab (*n* = 12), capecitabine ± bevacizumab (*n* = 5), FOLFOXIRI (folinic acid + 5-FU + irinotecan + oxaliplatin) ± bevacizumab (*n* = 2) and trifluridine/tipiracil + bevacizumab (*n* = 1). In ten of these patients with prior systemic treatment, chemoradiation was applied. Median time between previous systemic treatment and fecal sample collection was 686 days (IQR = 813 days). In addition, 47.7% of the patients used prophylactic or therapeutic antibiotics in the year before inclusion, with a mean time of 113 days (SD = 103) between the last antibiotic use and T1 (Table 1). Thirty-two patients (72.7%) were current or past smokers.

**Table 1:** Baseline characteristics of the current study population (*n* = 44)

Clinical characteristics	
Age–years	Median (IQR) 74.5 (13)
Male gender	*n* (%) 32 (72.7%)
BMI—kg/m^2^	mean (SD) 27.2 (5.1)
Co-treatment with bevacizumab (7.5 mg/kg)	*n* (%) 32 (72.7%)
Sidedness tumor	*n* (%)
Left-sided	31 (70.5%)
Right-sided	12 (27.3%)
Missing	1 (2.3%)
Number of metastatic sites	*n* (%)
1	12 (27.3%)
2	19 (43.2%)
3 4	10 (22.7%) 2 (4.5%)
7	1 (2.3%)
Colostomy in situ	*n* (%) 14 (31.8%)
*Prior treatments*	
Prior systemic treatment	*n* (%) 20 (45.5%)
Antibiotic use last year	*n* (%) 21 (47.7%)
Colorectal surgery in the past	*n* (%) 37 (84.1%)
Type of colorectal surgery	*n* (%)
Rectum resection	16 (36.4%)
Sigmoid resection	7 (15.9%)
Hemicolectomy left	2 (4.5%)
Extended hemicolectomy left	1 (2.3%)
Hemicolectomy right	7 (15.9%)
Other	1 (2.2%)
Unknown	3 (6.8%)

### Tumor response and dose adjustments during three cycles of capecitabine

From the 44 patients included, tumor response according to the RECIST criteria could be evaluated in 42 patients (95.45%). None of these patients showed complete response, while six patients (14.3%) had a partial response. Stable disease was found in 31 patients (73.8%) and five patients (11.9%) showed progressive disease. In 29 out of 43 patients (67.4%), the starting dose of capecitabine was not adjusted during the study period. In six patients (13.9%), the dose needed to be reduced, while it was increased in seven patients (16.3%). In one patient (2.3%), the dose was reduced in cycle 2 due to reduced thrombocytes and back to the starting dose in cycle 3. Reasons for dose reductions were impaired renal function (*n* = 1), hand–foot syndrome (*n* = 4) or cytopenia (*n* = 1). Dose increases occurred because these patients started with a reduced dose in cycle 1, which could later be increased due to good tolerance. After completion of the study, 36 patients (81.8%) continued with the fourth cycle of capecitabine.

### Nutritional status, physical performance and the prevalence of chemotherapy-induced toxicity during three cycles of capecitabine

During three cycles of capecitabine, the risk for malnutrition (MUST score) did not change significantly in the current study population (*p* = 0.127) (Table S3). KPS scores, which are patient-reported measures of physical performance status, decreased significantly at T2 (median = 80, IQR = 20, *p* = 0.02) and T3 (median = 80, IQR = 29, *p* = 0.021) when compared to T1 (median = 90, IQR = 15) (Table S3).

**Fig. 1:**
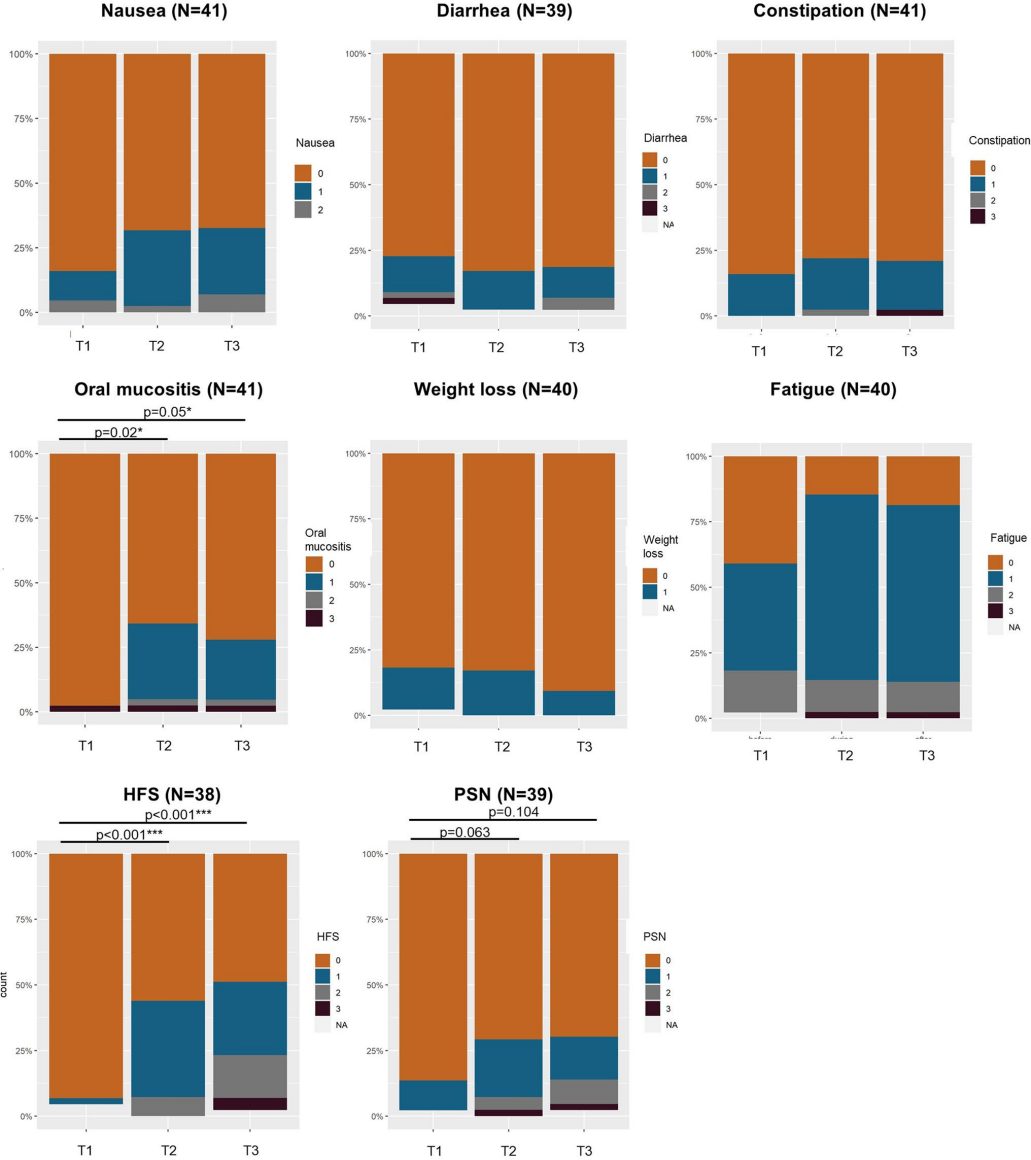
Symptoms related to chemotherapy-induced toxicity before (T1), during (T2) and after (T3) three cycles of capecitabine. The number of complete cases (= individuals who have values for all three time points) is given per variable. Results from post hoc analysis (adjusted p-values) are indicated for variables that showed significant differences according to Friedman’s ANOVA. HFS = hand–foot syndrome. PSN = peripheral sensory neuropathy. NA = missing values

Regarding chemotherapy-induced toxicity, Friedman’s ANOVA showed that the prevalence of oral mucositis (*p* < 0.001), the hand–foot syndrome (HFS) (*p* < 0.001) and peripheral sensory neuropathy (*p* = 0.039) increased during three cycles of capecitabine, as illustrated in Fig. 1 and Table S4. Post hoc analysis revealed that oral mucositis and HFS were significantly more prevalent at T2 and T3 compared to T1 (Fig. 1). The increase in peripheral sensory neuropathy was no longer significantly different after post hoc analysis with Bonferroni correction (Fig. 1). The prevalence of nausea (*p* = 0.118), diarrhea (*p* = 0.368), unintended weight loss (*p* = 0.236), constipation (*p* = 0.558) and fatigue (*p* = 0.146) did not significantly increase during capecitabine treatment as compared to baseline (Fig. 1 and Table S4).

### Levels of valerate and caproate decreased during capecitabine, while levels of the other SCFA and BCFA remained unchanged

**Fig. 2:**
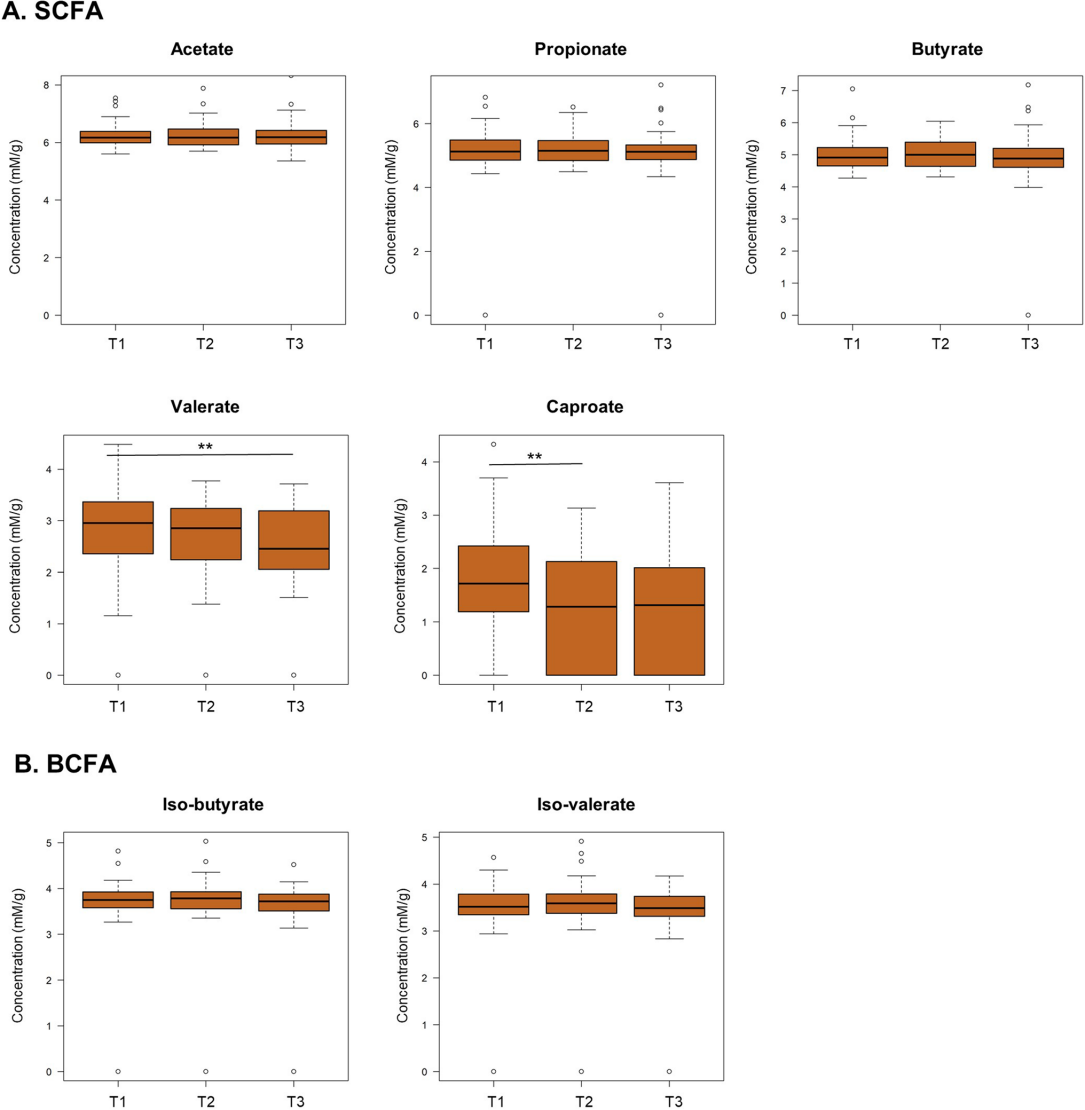
Changes in fecal SCFA and BCFA concentrations (mM/g dry weight; log-transformed (log1p)) during three cycles of capecitabine. Significant differences according to paired Wilcoxon signed-rank test with Bonferroni correction are indicated with asterisks (**p < 0.01)

In the current study population, fecal levels of acetate were highest across all time points (median = 478.42 mM/g, IQR = 242.41 mM/g), followed by propionate (median = 166.39 mM/g, IQR = 92.88 mM/g), butyrate (median = 134.52 mM/g, IQR = 88.96 mM/g), iso-butyrate (median = 40.91 mM/g, IQR = 14.99 mM/g), iso-valerate (median = 33.74 mM/g, IQR = 14.4 mM/g), valerate (median = 14.78 mM/g, IQR = 16.46 mM/g) and caproate (median = 3.30 mM/g, IQR = 7.94 mM/g). In general, we observed considerable inter-individual variability of fecal SCFA and BCFA levels. Friedman’s ANOVA indicated that fecal concentrations of the SCFA valerate (*x*^2^ = 10.74, *p* = 0.005) and caproate (*x*^2^ = 8.842, *p* = 0.012) decreased significantly during three cycles of capecitabine. Post hoc analysis with Bonferroni correction showed that valerate concentrations were significantly different between T1 and T3 (*p*_adjusted_ = 0.001), while caproate concentrations reduced significantly between T1 and T2 (*p*_adjusted_ = 0.008) (Fig. 2). Fecal levels of the SCFA acetate (*x*^2^ = 1.513, *p* = 0.469), propionate (*x*^2^ = 1.135, *p* = 0.567) and butyrate (*x*^2^ = 0.162, *p* = 0.922) as well as of the BCFA iso-butyrate (*x*^2^ = 0.676, *p* = 0.713) and iso-valerate (*x*^2^ = 1.401, *p* = 0.496) were not significantly different between T1, T2 and T3 (Fig. 2).

These results were confirmed using linear mixed models based on log-transformed data, also indicating a significant reduction of valerate and caproate during capecitabine (95% confidence intervals: valerate: − 0.404; − 0.081 and caproate: − 0.444; − 0.027) and no reduction of the other SCFA and BCFA (Table S5).

### Prior treatment, bevacizumab co-treatment or the necessity for dose adjustments had no major impact on fecal levels of SCFA and BCFA

Cross-sectional analyses revealed that neither prior chemotherapy nor antibiotic administration before T1 caused statistically significant differences in fecal SCFA or BCFA concentrations compared to patients without prior treatment (Table S6). In addition, co-treatment with bevacizumab did not impact fecal levels of SCFA or BCFA at sampling time points T2 and T3 in the present study population (Table S6). Concerning capecitabine dose adjustments, it was found that fecal SCFA and BCFA levels at T1, T2 and T3 did not differ between patients with or without dose adjustments during three cycles of capecitabine (Table S6). Similarly, fecal SCFA and BCFA levels were not different between patients who did or did not continue with the fourth cycle of capecitabine after the study period (Table S6).

### Baseline BCFA iso-butyrate was associated with tumor response

It was hypothesized that higher fecal SCFA levels would be associated with a better tumor response during three cycles of capecitabine. Cross-sectional analysis with the Kruskal–Wallis test indicated that fecal levels of all SCFA as well as of the BCFA iso-valerate were similar among patients with progressive disease, stable disease or partial response at all time points (Table S6). However, fecal levels of the BCFA iso-butyrate were found to be significantly different between these groups (*p* = 0.014) at baseline (T1). Post hoc analysis by means of Dunn’s test with Bonferroni correction showed that iso-butyrate was significantly lower in the feces of patients with partial response compared to patients with stable disease (*p*_adjusted_ = 0.017) or progressive disease (*p*_adjusted_ = 0.043) (Figure S2).

**Fig. 3:**
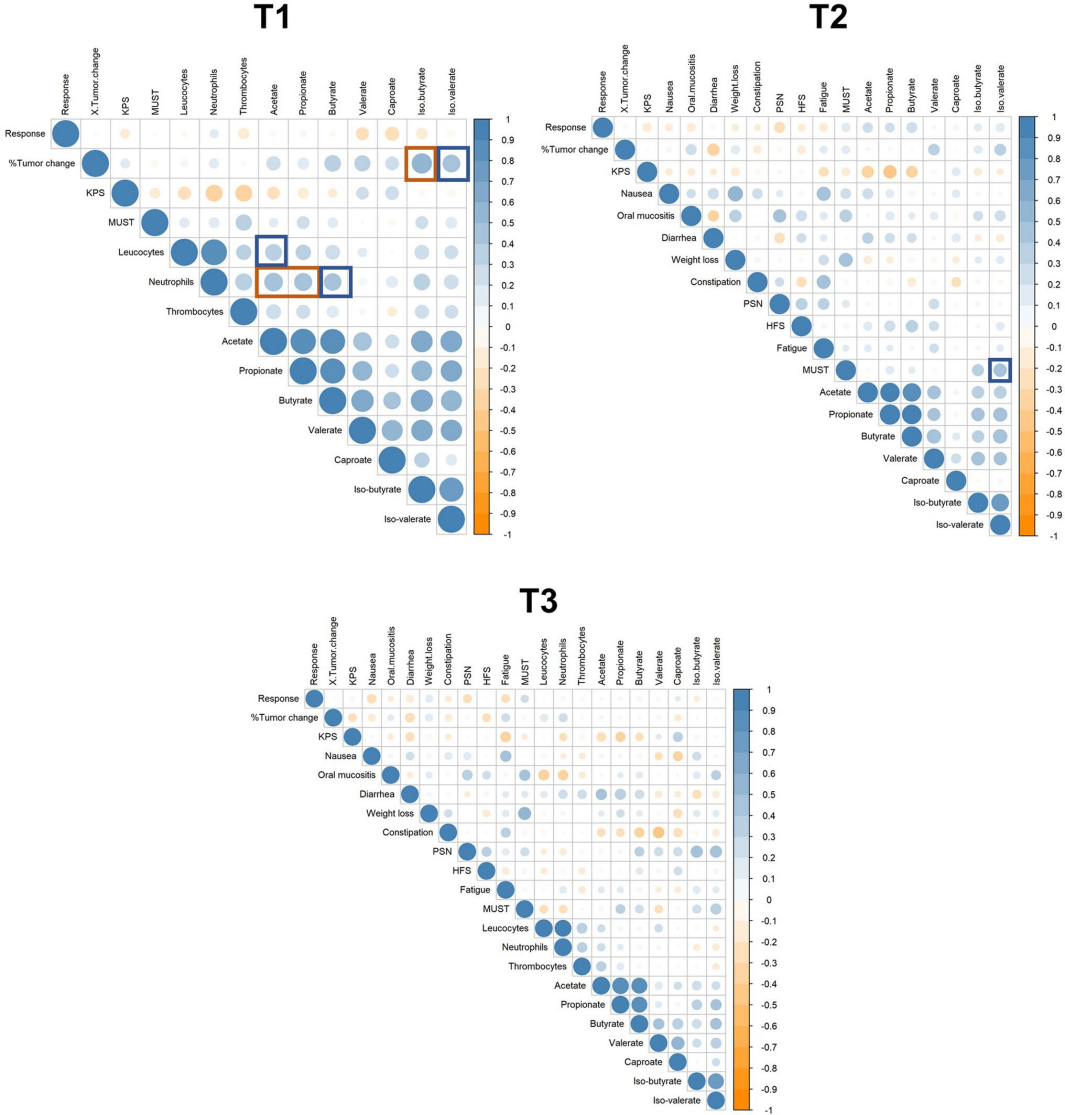
Spearman correlations between SCFA, BCFA and clinical as well as blood inflammatory parameters at different sampling time points (T1, T2 and T3). Significance was assessed for associations involving SCFA/BCFA only, not for potential relations between the other parameters. Significant correlations (*p* < 0.05) are marked with orange boxes, and correlations with a trend toward significance (*p* > 0.05 but < 0.07) are marked with blue boxes

Furthermore, correlation analysis revealed that fecal levels of iso-butyrate were positively correlated with tumor size change (%) (rho = 0.550, *p*_adjusted_ = 0.005, Figs. 3 and S3) at T1. Fecal levels of iso-valerate tended to be associated with tumor size change (%) at this time point (rho = 0.421, *p*_adjusted_ = 0.060, Figs. 3 and S3). Fecal SCFA and BCFA concentrations at T2 or T3 were not associated with tumor size change.

### Fecal SCFA and BCFA were not significantly correlated with nutritional status, physical performance or chemotherapy-induced toxicity

It was hypothesized that higher SCFA levels would be associated with better nutritional status (as assessed by the MUST score), increased physical performance (as assessed by KPS), as well as with less chemotherapy-induced toxicity.

Correlation analysis revealed that none of the SCFA or BCFA concentrations were significantly associated with the MUST or KPS scores at T1, T2 or T3 in the present study population. There was a statistically nonsignificant trend toward a positive association between iso-valerate and MUST at T2 (rho = 0.433, *p*_adjusted_ = 0.066, Figs. 3 and S3). In addition, no statistically significant correlations were found between fecal SCFA and BCFA concentrations and chemotherapy-induced toxicity during (T2) or after (T3) three cycles of capecitabine.

In order to evaluate the reliability of patient-reported diarrhea scores, we also investigated whether patient-reported diarrhea was associated with the sample dry weight as assessed in our laboratories. At T1 and T3, a higher score for diarrhea was significantly associated with lower sample dry weight (T1: rho = -0.391, *p* = 0.010; T3: rho = − 0.489, *p* = 0.002, Figure S4). This negative correlation was also present, but not statistically significant at T2 (rho = − 0.318, ip = 0.055, Figure S4).

### Baseline SCFA correlated with blood neutrophil counts

Furthermore, it was hypothesized that higher fecal levels of SCFA would be associated with reduced systemic inflammation, which would be reflected in reduced levels of the blood counts of leukocytes, neutrophils and thrombocytes (in 10^9^/l). Correlations with blood inflammatory parameters were tested at T1 and T3 only since no blood was drawn in close proximity to T2.

At T1, higher fecal levels of acetate (rho = 0.469, *p*_adjusted_ = 0.021), as well as propionate (rho = 0.428, *p*_adjusted_ = 0.043) were significantly correlated with an increased count of blood neutrophils (Figs. 3 and S3). In addition, also butyrate tended to be positively correlated with neutrophils (rho = 0.405, *p*_adjusted_ = 0.061, Figs. 3 and S3), but this association did not reach statistical significance. Additionally, there was a nonsignificant trend toward a positive correlation between acetate and leukocytes (rho = 0.378, *p*_adjusted_ = 0.061, Figs. 3 and S3) at T1. At T3, we did not identify significant correlations between fecal SCFA or BCFA and blood inflammatory parameters.

### Associations between SCFA, BCFA and bacterial abundances

**Fig. 4:**
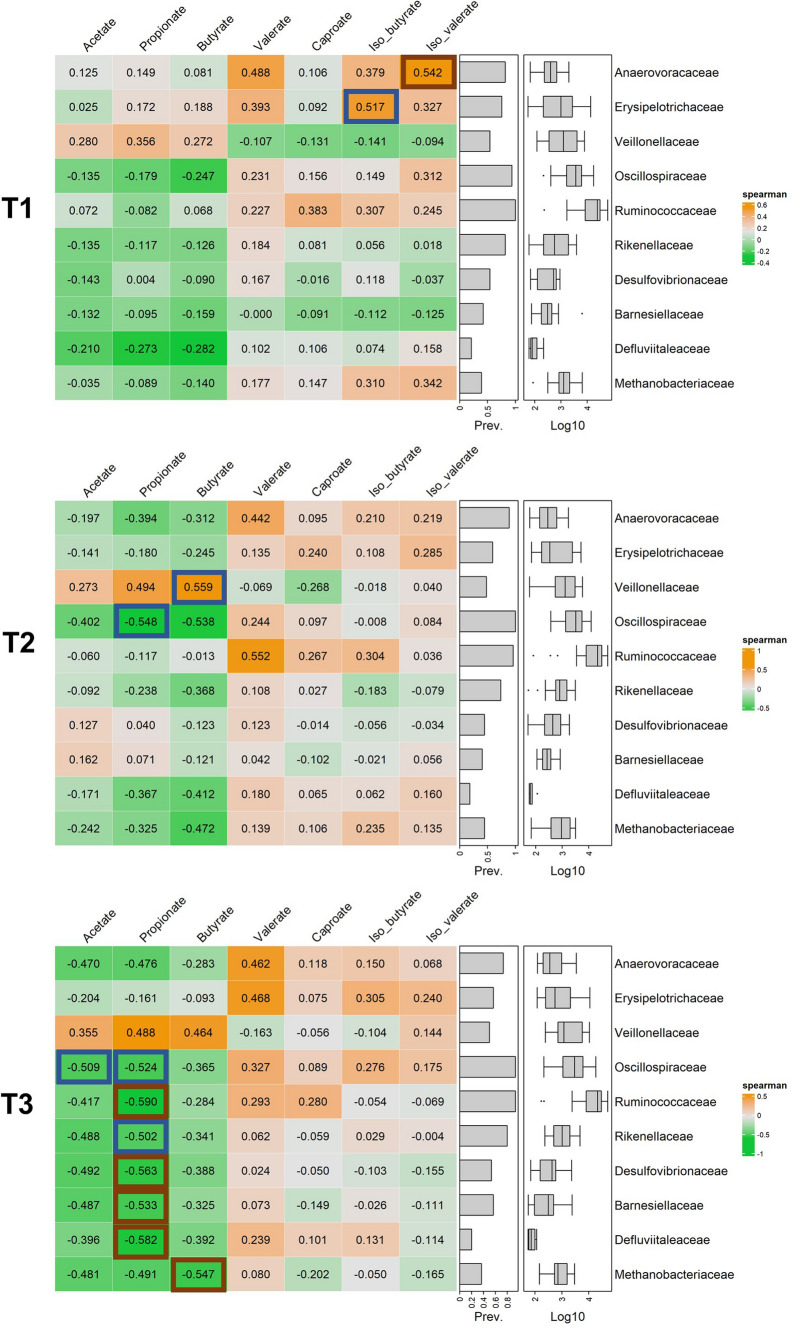
Correlation heatmaps with Spearman correlation coefficients for the correlations between fecal levels of SCFA and BCFA and the relative abundance of bacterial taxa on family level. All taxa which showed associations with SCFA/BCFA at one of the time points (*p*-value < 0.07) were included. Prevalence and log10 abundance are depicted for each taxon. Significant correlations (*p* < 0.05) are marked with orange boxes, and correlations with a trend toward significance (*p* > 0.05 but < 0.07) are marked with blue boxes

In a subgroup of patients (*n* = 32, 89 samples), we also related fecal levels of SCFA and BCFA to the relative abundance of bacterial families, as assessed by 16S rRNA V4 amplicon sequencing. At T1, iso-valerate correlated significantly and positively with Anaerovoracaceae (*p*_adjusted_ = 0.039). In addition, there was a positive correlation between iso-butyrate and Erysipelotrichaceae, which was not statistically significant (*p*_adjusted_ = 0.057) (Fig. 4). At T2, butyrate tended to be positively correlated with Veillonellaceae (*p*_adjusted_ = 0.068), while propionate tended to be negatively correlated with Oscillospiraceae (*p*_adjusted_ = 0.068) (Fig. 4). Most associations between SCFA/BCFA and the relative abundance of bacterial taxa were identified at T3 (Fig. 4). Again, Oscillospiraceae tended to be negatively associated with propionate (*p*_adjusted_ = 0.055) and at this time point also with acetate (*p*_adjusted_ = 0.067). Furthermore, statistically significant negative associations were found between propionate and Ruminococcaceae (*p*_adjusted_ = 0.022), Desulfovibrionaceae (*p*_adjusted_ = 0.029), Barnesiellaceae (*p*_adjusted_ = 0.045) and Defluviitaleaceae (*p*_adjusted_ = 0.024) as well as a nonsignificant association between propionate and Rikenellaceae (*p*_adjusted_ = 0.067). Fecal levels of butyrate were negatively correlated with the relative abundance of Methanobacteriaceae (*p*_adjusted_ = 0.036).

## Discussion

The current study indicated that fecal levels of the SCFA valerate and caproate decreased significantly during three cycles of capecitabine in patients with metastatic or unresectable CRC. Furthermore, we showed that baseline fecal levels of the BCFA iso-butyrate were associated with tumor response. Nutritional status, physical performance and chemotherapy-induced toxicity were not statistically significantly associated with SCFA or BCFA. Concerning systemic inflammation, it was found that baseline SCFA correlated positively with blood neutrophil counts. Lastly, fecal levels of SCFA and BCFA were associated with relative abundance of different bacterial families at the three time points under investigation.

Interestingly, we identified a reduction of fecal valerate and caproate levels during capecitabine treatment, while concentrations of the more common SCFA acetate, propionate and butyrate remained stable. A possible explanation is that acetate, propionate and butyrate are produced by a wider range of bacterial species [17]. Consequently, SCFA production could potentially be taken over by other gut bacteria that fill the niche if the abundance of dominant SCFA producers would change during capecitabine treatment. A similar mechanism has been already described for antibiotics [43]. On the other hand, our results suggest an effect of capecitabine treatment on fecal levels of valerate and caproate. Concentrations of these SCFA are generally lower compared to the other SCFA, and their potential physiological roles are currently poorly understood. Valerate is produced by some *Clostridium* species via different mechanisms and has been shown to inhibit the growth of the pathogenic *Clostridioides difficile* [17, 44]. In addition, Hinnebusch et al. showed that valerate, next to propionate and butyrate, caused histone hyperacetylation and growth inhibition in human carcinoma cells [45]. This suggests that the observed decrease during capecitabine treatment could also be of relevance for tumor response. The exact cause(s) of this valerate reduction is unknown, but one possible explanation is that capecitabine might impact the abundance of valerate-producing bacteria. Alternatively, capecitabine might interfere with pathways or intermediate metabolites involved in valerate metabolism [46, 47]. In any case, the molecular interactions between capecitabine, valerate and caproate require further investigation, for instance by future in vitro incubation experiments.

Since a significant proportion of the patients in our study population received chemotherapy (> 1 month) or antibiotic treatment (> 3 months) before inclusion, we also assessed whether this had an impact on fecal SCFA/BCFA levels. In contrast to the previous literature describing considerable chemotherapy-induced changes in gut microbiota composition (e.g., [2, 28, 29, 48]) and detrimental effects of antibiotics (49), we did not identify significant differences between those groups. This suggests that the chosen washout periods were sufficient to prevent the confounding effects of prior treatments. However, the complexity of the current study population should be taken into consideration. In view of extensive and complex medical histories, we cannot rule out that previous therapies disturbed gut barrier function and thereby SCFA/BCFA absorption into the blood. Impaired absorption might lead to increased fecal excretion, while actual production might be constant or even reduced. Due to a lack of adequate and noninvasive alternatives, fecal SCFA and BCFA are used as markers for the luminal SCFA/BCFA content in this study and should be interpreted accordingly.

Additionally, we hypothesized that, if higher fecal SCFA would reflect higher production, it would be associated with better tumor response. This was expected based on the earlier described anti-carcinogenic effects of particularly butyrate [11, 50], but also valerate and propionate [45]. While butyrate is the preferred energy substrate for normal colonocytes, cancer cells preferably consume glucose. Consequently, butyrate is accumulated in cancer cells and can act as an HDAC inhibitor there, modulating cell proliferation, apoptosis and differentiation [50]. Surprisingly, we did not find an association between SCFA and tumor response in our patient cohort. On the other hand, the baseline values of the BCFA iso-butyrate were significantly lower in patients who showed partial response (at least − 30% tumor size change) and were also correlated with tumor size change. This suggests that baseline BCFA levels should be further evaluated as potential factor to predict tumor response in these patients. Furthermore, it might be hypothesized that increased fecal BCFA could be a sign of increased amino acid catabolism in these patients, since BCFA can also be produced by gut bacteria through branched-chain amino acid digestion [16, 17]. In patients with advanced CRC, increased amino acid catabolism could potentially be caused by cancer cachexia. Cancer cachexia is a multifactorial metabolic syndrome, which is characterized by increased protein degradation and loss of muscle mass and also negatively affects treatment outcomes [51, 52]. Therefore, it might be beneficial to also include markers of cancer cachexia (e.g., exact weight loss, body composition) in future studies and to further explore the association between baseline BCFA and tumor response. More knowledge on this association and the potential predictive value of baseline BCFA could be of great relevance to identify patients who are at risk for a suboptimal tumor response already before start of the treatment.

In the current research population, we did not identify statistically significant correlations between fecal levels of SCFA/BCFA and nutritional status, physical performance or chemotherapy-induced toxicity. This was unexpected regarding the known beneficial effects of SCFA [4, 9] and not in line with a previous study reporting an association between the SCFA-producing *Eubacterium hallii* and fatigue [53]. In contrast to the earlier described anti-inflammatory effects of SCFA (e.g., [6, 9]), higher fecal SCFA were associated with increased concentrations of blood inflammatory markers in our patient population, which could be caused by disturbed SCFA absorption, as described above. As a next step, we investigated associations between SCFA, BCFA and the abundance of bacterial taxa. Interestingly, iso-butyrate, which seemed to have negative effects on tumor response in our patient population, tended to be associated with the relative abundance of Erysipelotrichaceae at T1. It has been previously described that this family was enriched in CRC and might be associated with lipid metabolism and inflammation [54].

There are some methodological limitations inherent to the current study, which should be taken into account when interpreting the results. First, the current sample size is relatively small and fecal SCFA/BCFA levels varied consistently between patients. Consequently, the current study should be seen as a pilot study, providing first indications concerning the role of SCFA/BCFA during capecitabine treatment. Furthermore, it should be noted that the patients harbored diverse and complex medical histories as well as different living environments and dietary habits, which could have confounding effects on SCFA and BCFA levels and might also contribute to the observed large heterogeneity. Particularly dietary fiber intake is considered to have a relevant role here because non-digestible carbohydrates are the precursors of SCFA [9]. Another potential confounding factor that was not assessed in the current research is gut transit time [55]. In addition, it should be noted that fecal and blood samples were not always collected on the same day, since blood sample analysis was part of standard care and depended on individual treatment schedules.

To the best of our knowledge, this is the first clinical study evaluating fecal SCFA and BCFA in patients with metastatic or unresectable CRC during treatment with capecitabine. The current study provides first indications that SCFA and BCFA might be of relevance during treatment with capecitabine and should also be considered in future studies. By exploring various correlations in a clinical setting, we provide a set of different points of attention for future studies and hope to stimulate a new understanding of the role of SCFA/BCFA during chemotherapy. However, the gut microbiota also produces numerous other metabolites with diverse functions, for instance phenolic acids, secondary bile acids or polyamines [1]. Therefore, future research should also investigate the net metabolic output as well as the metabolic capacity of the gut microbiota by metabolomics or metagenomic sequencing. Since the gut microbiota is not an isolated organism but a whole ecosystem with numerous interactions, it will be pivotal, but challenging, to elucidate the complex and diverse mutual interactions between gut bacteria, their metabolites and chemotherapy. Furthermore, our data suggest that more attention should be given to valerate and caproate. Although only present in low concentrations, these SCFA could potentially have relevant physiological roles, especially in dysbiotic and pro-inflammatory conditions during chemotherapy. Similarly, the role of BCFA in tumor response and underlying molecular mechanisms should be explored further. In line with this, future research could examine whether baseline BCFA could be used to predict tumor response to capecitabine, as suggested by our results. Furthermore, the association between SCFA and systemic inflammation in CRC needs further investigation. To assess possible malabsorption of SCFA, blood SCFA concentrations should also be assessed in future studies. Likewise, it might be beneficial to include markers of gastrointestinal inflammation (e.g., fecal calprotectin) and gut transit time [55, 56].

## Conclusions

Altogether, the present study provided the first indications for a role of SCFA and BCFA during treatment with capecitabine as well as implications and recommendations for further research. More knowledge on the exact roles of these gut microbiota-derived metabolites will contribute to the evidence-based design of interventions targeting the gut microbiota and/or SCFA/BCFA production during chemotherapy.

### Supplementary Information

Below is the link to the electronic supplementary material.

Supplementary file1 (PDF 1013 kb)

## Data Availability

Sequencing data were submitted to Qiita and deposited in the European Nucleotide Archive (ENA). The accession code is: ERP143365. Additional data used and/or analyzed during the current study are available from the corresponding author on reasonable request.

## Abbreviations

5-FU: 5-Fluorouracil
BCFA: Branched-chain fatty acids
CRC: Colorectal cancer
CTCAE: Common Terminology Criteria for Adverse Events
GC–MS: Gas chromatography—mass spectrometry
HDAC: Histone deacetylases
ICTRP: International Clinical Trial Registry Platform
KPS: Karnofsky Performance Score
mCRC: Metastatic colorectal cancer
MUMC+: Maastricht University Medical Center
MUST: Malnutrition Universal Screening Tool
RECIST: Response Evaluation Criteria in Solid Tumours
SCFA: Short-chain fatty acids
