# Supplementary material for: Fecal levels of SCFA and BCFA during capecitabine in patients with metastatic or unresectable colorectal cancer
Source: Clin Exp Med. 2023 Apr 7;23(7):3919–33. doi: 10.1007/s10238-023-01048-7 (PMC10618330; doi:10.1007/s10238-023-01048-7)
Supplement: Supplementary file 1 — Supplementary file1 (PDF 1013 kb) [file 10238_2023_1048_MOESM1_ESM.pdf]

Supplementary tables and figures

Table S1: *Scoring of toxicity according to CTCAE criteria. Patients were asked to select the score describing the current situation at the time of fecal sample collection*

Symptom	Score	Explanation	Symptom	Score	Explanation
Nausea	0	Not at all	Unintentional weight loss past 3-6 months	0	Not at all (less than 5%)
	1	Less appetite		1	Moderate weight loss (between 5 and 10%)
	2	Less food intake		2	Severe weight loss (between 10 and 20%)
	3	Insufficient food intake		3	Very severe weight loss (>20%)
Vomiting	0	Not at all	Constipation	0	Not at all
	1	1-2 times per 24 hours		1	Occasional complaints
	2	3-5 times per 24 hours		2	Persistent complaints
	3	6 or more times per 24 hours		3	Necessary to remove stool by hand
	4	life threatening, acute intervention needed		4	Life threatening
Fever	0	Not at all	Peripheral sensory neuropathy	0	Not at all
	1	38°C – 39°C		1	Minimal complaints
	2	39,1°C – 40°C		2	Moderate complaints
	3	More than 40°C, less than 24 hours		3	Severe complaints
	4	More than 40°C, more than 24 hours		4	Life threatening
Diarrhea (patients without stoma)	NA	Not applicable	Diarrhea (Patients with stoma)	NA	Not applicable
	0	Not at all		0	Not at all
	1	Compared to normal an increase of <4 times stool per day		1	Compared to normal a minimal increase
	2	Compared to normal an increase of 4-6 times stool per day		2	Compared to normal a moderate increase
	3	Compared to normal an increase of >7 times stool per day		3	Compared to normal a severe increase
	4	Life threatening, acute intervention needed		4	Life threatening
Fatigue	0	Not at all	Hand-foot syndrome	0	Not at all
	1	Fatigue which decreases after rest		1	Skin changes or skin inflammation without pain
	2	Fatigue which does not decrease after rest: limited possibility to normal daily functioning		2	Skin changes with pain: with limited possibility to normal daily functioning
	3	Fatigue which does not decrease after rest: limited possibility to self-care		3	Severe skin changes with pain with limited possibility to self-care
Oral mucositis	0	Not at all	Hair loss	0	Not at all
	1	No complaints or mild complaints		1	Hair loss <50%
	2	Moderate pain		2	Hair loss >50%
	3	Severe pain			
	4	Life threatening			

Table S2: *Calculation of the %tumor change before and after three cycles of capecitabine and classification of tumor response according to the RECIST categories*

**Calculation of %tumor size change**	
SLD before	Sum longest diameter of the target lesion before start of treatment with capecitabine
SLD after	Sum longest diameter of the target lesion after three cycles of capecitabine
Formula	$\frac{(SLD\ after - \ SLD\ before)}{SLD\ before} x\ 100\%$
**RECIST categories**	
Complete response	Disappearance of all target lesions
Partial response	At least 30% decrease in the sum of the target lesions
Progressive disease	At least 20% increase in the sum of target lesions
Stable disease	Small changes that did not meet above criteria

Table S3: *MUST scores and Karnofsky Performance Scores (KPS) before (T1), during (T2) and after (T3) three cycles of capecitabine. Percentages are calculated based on valid measurements (missings are excluded).*

	**T1**<br>n(%)	**T2**<br>n(%)	**T3**<br>n(%)
MUST – low risk	34 (77.3%)	34 (82.9%)	39 (92.8%)
MUST – medium risk	7 (15.9%)	6 (14.6%)	1 (2.4%)
MUST – high risk	3 (6.8%)	1 (2.4%)	2 (4.8%)
	**T1**<br>median (IQR)	**T2**<br>median (IQR)	**T3**<br>median (IQR)
KPS	90 (15)	80 (20)	80 (20)

Table S4: *Number of patients suffering from chemotherapy-induced toxicities before (T1), during (T2) and after (T3) three cycles of capecitabine. Percentages are calculated based on valid measurements (missings are excluded)*

Gastrointestinal complications			
	**T1**<br>n(%)	**T2**<br>n(%)	**T3**<br>n(%)
Nausea			
Grade 0	37 (84.1%)	28 (68.3%)	29 (67.4%)
Grade 1	5 (11.4%)	12 (29.3%)	11 (25.6%)
Grade 2	2 (4.5%)	1 (2.4%)	3 (7.0%)
Diarrhea			
Grade 0	34 (80.9%)	34 (85.0%)	35 (83.3%)
Grade 1	6 (14.3%)	6 (15.0%)	5 (11.9%)
Grade 2	1 (2.4%)	-	2 (4.8%)
Grade 3	1 (2.4%)	-	-
Constipation			
Grade 0	37 (84.1%)	32 (78.0%)	34 (79.1%)
Grade 1	7 (15.9%)	8 (19.5%)	8 (18.6%)
Grade 2	-	1 (2.4%)	1 (2.3%)
Oral mucositis			
Grade 0	43 (97.7%)	27 (65.9%)	31 (72.1%)
Grade 1	-	12 (29.3%)	10 (23.3%)
Grade 2	-	1 (2.4%)	1 (2.3%)
Grade 3	1 (2.3%)	1 (2.4%)	1 (2.3%)
Weight loss			
Grade 0	36 (83.7%)	34 (82.9%)	39 (90.7%)
Grade 1	7 (16.3%)	7 (17.1%)	4 (9.3%)
Peripheral sensory neuropathy			
Grade 0	38 (88.4%)	29 (70.7%)	30 (71.4%)
Grade 1	5 (11.6%)	9 (22.0%)	7 (16.7%)
Grade 2	-	2 (4.9%)	4 (9.5%)
Grade 3	-	1 (2.4%)	1 (2.4%)
Hand-foot-syndrome			
Grade 0	41 (97.6%)	23 (56.1%)	21 (50.0%)
Grade 1	1 (2.4%)	15 (36.6%)	12 (28.6%)
Grade 2	-	3 (7.3%)	7 (16.7%)
Grade 3	-	-	2 (4.8%)
Fatigue			
Grade 0	18 (41.9%)	6 (14.6%)	8 (18.6%)
Grade 1	18 (41.9%)	29 (70.7%)	29 (67.4%)
Grade 2	7 (16.3%)	5 (12.2%)	5 (11.6%)
Grade 3	-	1 (2.4%)	1 (2.3%)

Table S5: *Results from the linear mixed model testing the fixed effect of sampling timepoint, correcting for random effects produced by longitudinal sampling within patients*

**SCFA**	Estimate	Std. Error	t-value	2.5% - 97.5%
**Acetate**	0.00096	0.033	0.029	-0.065 - 0.067
**Propionate**	-0.06031	0.096	-0.629	-0.249 - 0.128
**Butyrate**	-0.1150	0.083	-1.388	-0.278 - 0.048
**Valerate**	-0.2423	0.082	-2.955	-0.404 – (-0.081)*
**Caproate**	-0.2362	0.106	-2.232	-0.444 – (-0.027)*

**BCFA**	Estimate	Std. Error	t-value	2.5% - 97.5%
**Iso-butyrate**	-0.1346	0.133	-1.012	-0.397 - 0.127
**Iso-valerate**	-0.05961	0.095	-0.627	-0.247 - 0.127

Table S6: *Cross-sectional differences in SCFA and BCFA concentrations*

**Dose adjustments during T1 – T3** (groups: no dose adjustments, dose reduction, dose increase, dose increase and reduction)									
Kruskal Wallis Test	**T1**			**T2**			**T3**		
	**X^2**	**df**	**p-value**	**X^2**	**df**	**p-Value**	**X^2**	**df**	**p-Value**
**Acetate**	3.6827	3	0.2978	1.7386	3	0.6284	5.3489	3	0.148
**Propionate**	3.296	3	0.3482	0.29991	3	0.96	4.5232	3	0.2102
**Butyrate**	2.9675	3	0.3967	0.76836	3	0.857	3.7203	3	0.2933
**Valerate**	1.378	3	0.7107	1.6757	3	0.6424	4.0907	3	0.2518
**Caproate**	1.7971	3	0.6156	6.227	3	0.1011	2.4859	3	0.4779
**Iso-butyrate**	2.7853	3	0.4259	2.6389	3	0.4507	2.2016	3	0.5316
**Iso-valerate**	4.1181	3	0.249	2.217	3	0.5286	2.9868	3	0.3937

**Tumor response** (groups: progressive disease, stable disease, partial response)									
Kruskal Wallis Test	**T1**			**T2**			**T3**		
	**X^2**	**df**	**p-value**	**X^2**	**df**	**p-value**	**X^2**	**df**	**p-value**
**Acetate**	0.506	2	0.777	2.566	2	0.277	0.059	2	0.971
**Propionate**	0.448	2	0.799	2.322	2	0.313	0.148	2	0.928
**Butyrate**	2.087	2	0.352	3.216	2	0.200	0.306	2	0.858
**Valerate**	4.633	2	0.099	0.316	2	0.854	0.343	2	0.842
**Caproate**	3.397	2	0.183	0.418	2	0.811	1.062	2	0.588
**Iso-butyrate**	8.544	2	0.014*	0.779	2	0.677	0.454	2	0.797
**Iso-valerate**	2.184	2	0.336	1.380	2	0.501	0.602	2	0.740

*significant result of Kruskal Wallis test, subsequently a post-hoc test Dunn's test with Bonferroni correction was performed:
stable disease vs. partial response: p.adjusted=0.017
stable disease vs. progressive disease: p.adjusted=1.000
partial response vs. progressive disease: p.adjusted=0.043

**Therapy continuation** (groups: continuation capecitabine (4th cycle), no continuation capecitabine)						
Mann Whitney U Test	**T1**		**T2**		**T3**	
	**W**	**p-value**	**W**	**p-value**	**W**	**p-value**
**Acetate**	133	0.834	82	1.0	101	0.955
**Propionate**	155	0.356	97	0.557	118	0.471
**Butyrate**	116	0.760	93	0.675	108.5	0.726
**Valerate**	114	0.711	86	0.897	133	0.192
**Caproate**	102	0.437	75	0.753	78.5	0.425
**Iso-butyrate**	166	0.193	76.5	0.812	112.5	0.612
**Iso-valerate**	155	0.348	84.5	0.948	130	0.235

**Systemic treatment before T1** (groups: no previous systemic treatment, previous systemic treatment)		
Mann Whitney U Test	**W**	**p-value**
**Acetate**	237	0.953
**Propionate**	230	0.825
**Butyrate**	242	0.972
**Valerate**	276	0.406
**Caproate**	276.5	0.393
**Iso-butyrate**	273	0.443
**Iso-valerate**	285.5	0.289

**Antibiotic use last year** (>3 months before inclusion, groups: no antibiotic use, antibiotic use)		
Mann Whitney U Test	**W**	**p-value**
**Acetate**	242	1.00
**Propionate**	245	0.944
**Butyrate**	249	0.871
**Valerate**	274	0.456
**Caproate**	197	0.298
**Iso-butyrate**	211	0.481
**Iso-valerate**	268	0.541

Table S6 continued: *Cross-sectional differences in SCFA and BCFA concentrations*

**Co-treatment with bevacizumab** (groups: bevacizumab co-treatment, no bevacizumab co-treatment)				
Mann Whitney U Test	**T2**		**T3**	
	**W**	**p-value**	**W**	**p-value**
**Acetate**	154	0.660	197	0.098
**Propionate**	133	0.832	176	0.327
**Butyrate**	128	0.708	180.5	0.260
**Valerate**	131	0.778	173	0.376
**Caproate**	95.5	0.123	137	0.805
**Iso-butyrate**	147.5	0.816	135	0.759
**Iso-valerate**	142	0.960	113	0.311

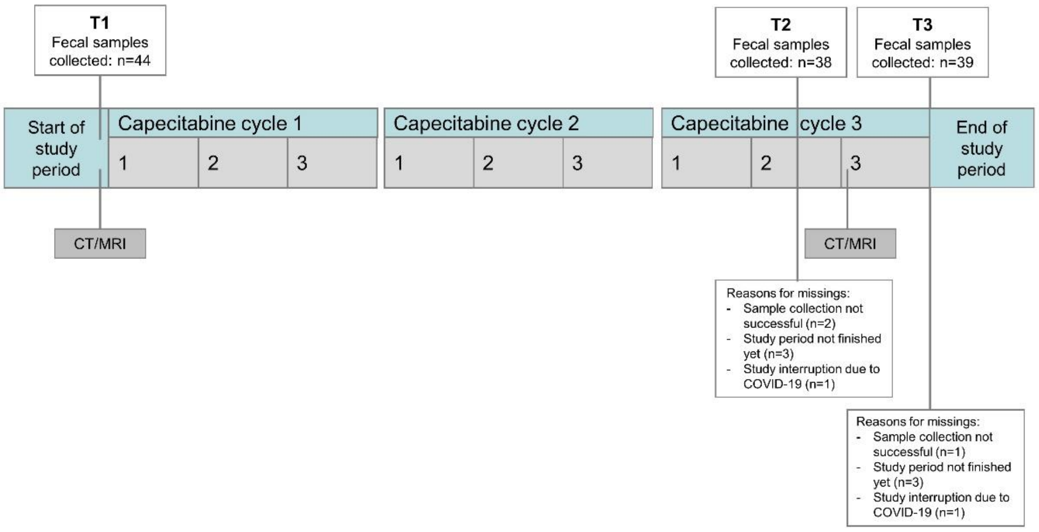

Figure S1: *Overview of study period and sampling timepoints.*

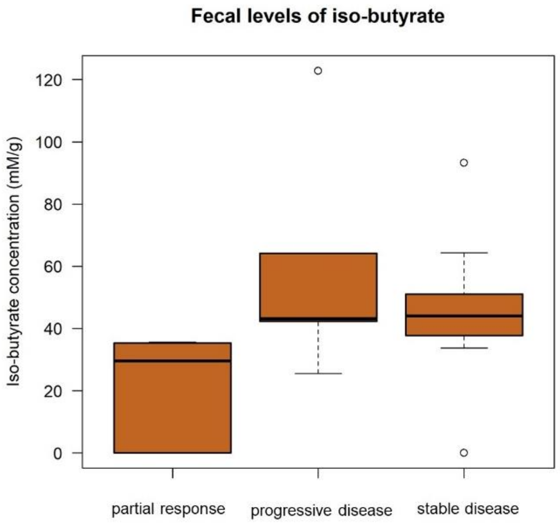

Figure S2: *Fecal levels of iso-butyrate at T1 were significantly lower in patients showing partial response compared to patients with stable disease ($p_{adjusted}$=0.017) or progressive disease ($p_{adjusted}$=0.043)*

A. Clinical parameters

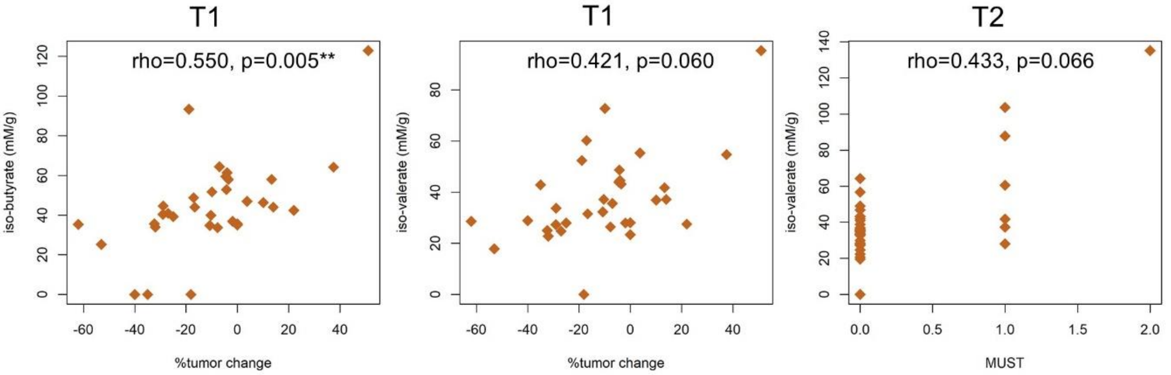

B. Blood inflammatory parameters

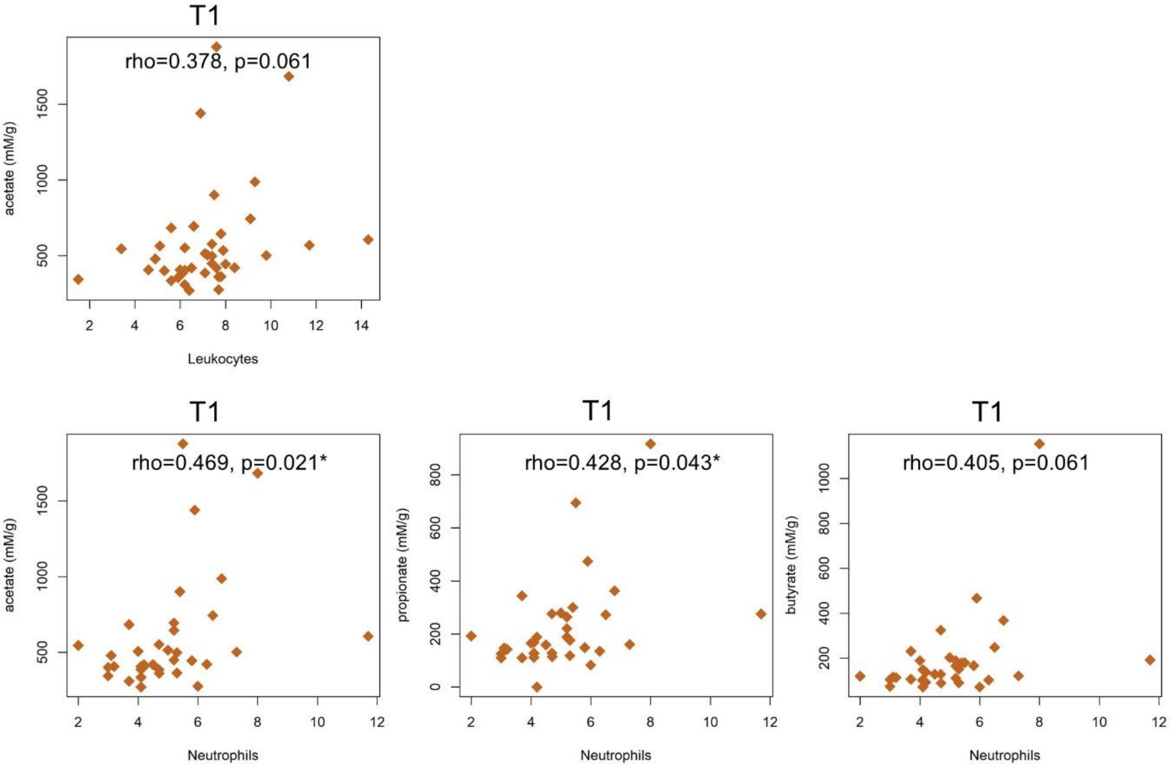

Figure S3: *Scatterplots for correlations between SCFA/BCFA and clinical as well as blood inflammatory parameters. Statistical significance is indicated with asterisks: *=p<0.05; **=p<0.01*

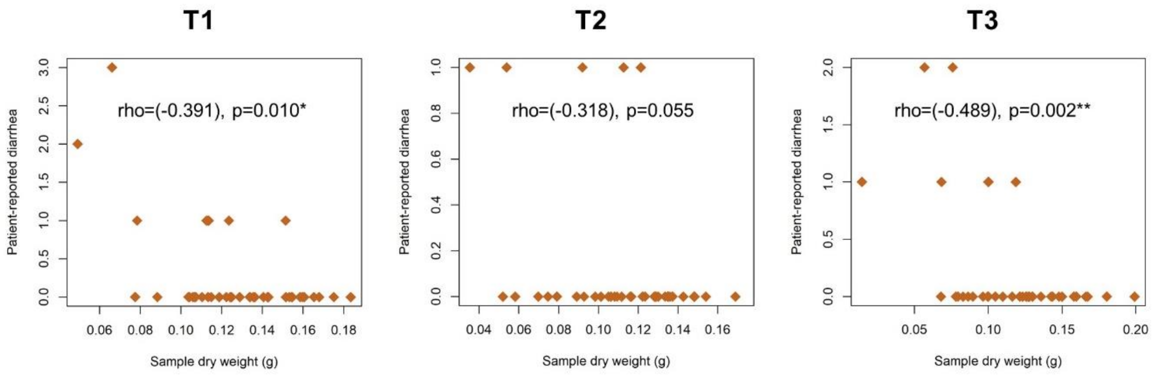

Figure S4: *Scatterplots for correlations between sample dry weight and patient-reported diarrhea. Statistical significance is indicated with asterisks: *=p<0.05; **=p<0.01*
